# Nondisruptive Full-Thickness Double-Row Repair Technique for Bursal-Sided Partial-Thickness Rotator Cuff Tears

**DOI:** 10.1016/j.eats.2025.103912

**Published:** 2025-11-10

**Authors:** Qiangqiang Li, Yuejian Ding, Yu Zhang, Jianghui Qin, Qing Jiang, Dongyang Chen

**Affiliations:** Division of Sports Medicine and Adult Reconstructive Surgery, Department of Orthopedic Surgery, Nanjing Drum Tower Hospital, State Key Laboratory of Pharmaceutical Biotechnology, Nanjing University, Nanjing, China; Branch of National Clinical Research Center for Orthopedics, Sports Medicine and Rehabilitation, Nanjing, China; and Jiangsu Key Laboratory of Molecular Medicine, Nanjing, China

## Abstract

A variety of surgical techniques have been described for managing bursal-sided partial-thickness rotator cuff tears (PTRCTs). A key consideration when treating these lesions is whether to complete the tear or to preserve the remaining intact fibers. Accordingly, current surgical strategies for bursal-sided PTRCTs include in situ and tear completion repair; each approach has its own limitations. We describe a nondisruptive full-thickness double-row repair technique, which captures the full thickness of the rotator cuff tendon while minimizing disruption to the preserved articular surface fibers. After full-thickness passage of the anchor sutures through the intact tendon assisted with a spinal needle, lateral-row fixation is achieved with a down-pressing anchor. This technique aims to enhance tendon healing and maximize restoration of the native tendon to its anatomic footprint in patients with bursal-sided PTRCTs.

Rotator cuff tears can be classified as full or partial thickness, with partial-thickness rotator cuff tears (PTRCTs) further categorized into bursal-sided, articular-sided, and intratendinous types.^1^ The reported prevalence of PTRCTs ranges from 13% to 37%.^2^^,^^3^ Among these tears, bursal-side PTRCTs represent a less common subset, accounting for approximately 9% to 18% of cases.^4^ Although the indication for surgical intervention in bursal-side PTRCTs remains ambiguous, arthroscopic repair is generally recommended for active patients experiencing persistent pain and weakness despite conservative treatment and for tears that involve more than 50% of the tendon thickness or measure greater than 2 cm in diameter.^5^

Surgical strategies for bursal-sided PTRCTs vary depending on whether the intact tendon fibers are preserved, favoring in situ repair, or intentionally sacrificed, favoring tear completion.^6^ In situ repair focuses on reattaching the torn fibers to the anatomic footprint while maintaining the integrity of the remaining tendon and its insertion. Although this technique effectively restores the anatomic footprint, it carries the risk of tension mismatch between the bursal and articular sides, potentially leading to postoperative stiffness and persistent discomfort due to over-tensioning of the repaired tissue.^7^^,^^8^ Alternatively, the tear completion technique involves converting the partial tear into a full-thickness tear prior to repair, which leads to unnecessary damage to healthy articular-side fibers and disruption of the native footprint structure.^5^ Furthermore, removing intact tendon tissue may result in mismatched tendon tension and length after repair, compromising functional recovery.^9^ Despite these differences, both techniques have shown comparable outcomes in terms of function and magnetic resonance imaging (MRI) findings.^6^^,^^10^ We advocate preserving as much of the intact tendon as possible when treating bursal-sided PTRCTs. Although several techniques have been developed to protect the articular-sided fibers during bursal-side repair, they often involve additional surgical portals and risk further damage to the remaining tendon.^9^^,^^11^^,^^12^ In response to these challenges, we introduce an approach that preserves the integrity of the intact articular fibers and ensures robust fixation of the full-thickness tendon layer.

## Surgical Technique

**Fig 1:**
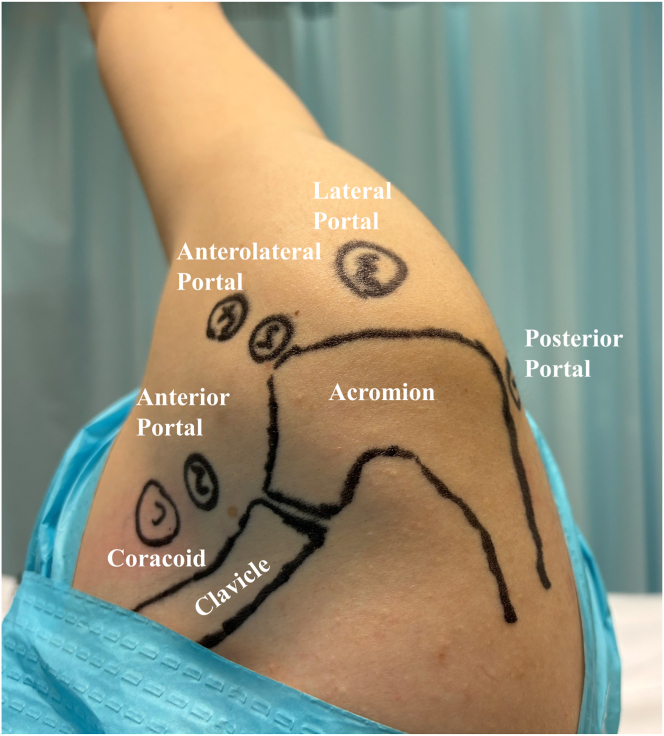
The operation is performed with the patient in the lateral decubitus position and the upper arm suspended at an abduction angle of 45°. The following portals are established: (1) standard posterior portal; (2) standard anterior portal; (3) lateral portal, in line with the posterior clavicle and 3 to 4 cm lateral to the acromion, used for visualization, debridement, and preparation of the rotator cuff footprint; and (4) anterolateral portal, typically 2 to 3 cm distal to the lateral edge of the acromion, used for acromioplasty, anchor insertion, and suture management.

**Fig 2:**
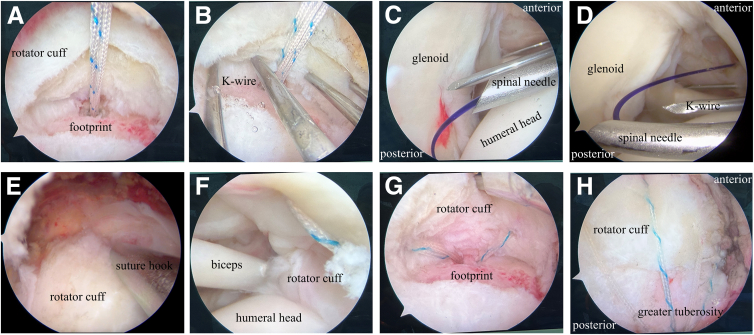
Partial-thickness bursal-sided rotator cuff tear of the right supraspinatus tendon in a 45-year-old male patient. (A) With the patient in the lateral decubitus position, arthroscopic imaging from the lateral viewing portal shows a partial-thickness bursal-sided rotator cuff tear of the right supraspinatus tendon after debridement. (B) Arthroscopic imaging of the right shoulder from the lateral viewing portal shows that the Kirschner wires are evenly passed through the residual tendon tissue. (C) Arthroscopic imaging of the right shoulder from the posterior viewing portal shows that a polydioxanone (PDS) suture is introduced percutaneously using an 18-gauge spinal needle. (D) Arthroscopic imaging from the posterior viewing portal shows that the PDS suture is retrieved through the anterior portal. (E) Arthroscopic imaging from the lateral viewing portal shows that a second PDS suture is introduced through a suture hook passing through the intact full-thickness tendon. (F) Arthroscopic imaging from the posterior viewing portal shows an anchor suture passing through the full-thickness tendon. (G) Arthroscopic imaging from the lateral viewing portal shows 4 anchor sutures evenly passing through the full-thickness tendon. (H) Arthroscopic imaging from the lateral viewing portal shows a subacromial view of the repair after the suture-bridge technique.

**Fig 3:**
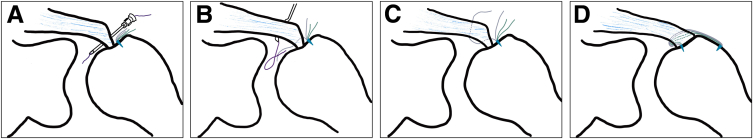
Surgical procedure. (A) A polydioxanone suture is introduced percutaneously using a spinal needle through the healthy tendon. (B) A suture hook is introduced via the anterolateral portal into the glenohumeral joint, passing through the intact full-thickness tendon. (C) An anchor suture is passing through the full-thickness tendon. (D) Suture-bridge fixation of supraspinatus tendon.

**Fig 4:**
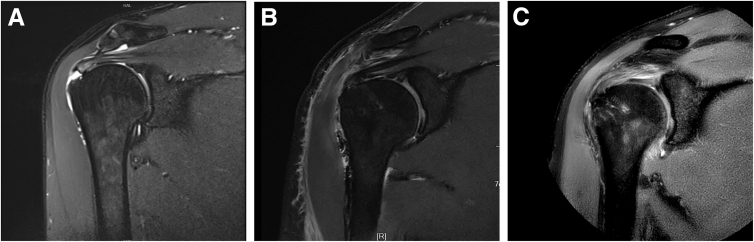
Partial-thickness bursal-sided rotator cuff tear of the right supraspinatus tendon in a 45-year-old male patient. (A) Oblique coronal magnetic resonance imaging (MRI) shows a partial-thickness bursal-sided rotator cuff tear preoperatively. (B) Oblique coronal MRI obtained at 1 day postoperatively. (C) Oblique coronal MRI obtained at 3 months postoperatively.

A standard posterior viewing portal is established (Fig 1), and the arthroscope is inserted into the glenohumeral joint (Video 1). An anterior portal is created through the rotator interval to address any intra-articular pathology. After the integrity of the articular-sided rotator cuff insertion is confirmed, the arthroscope is redirected into the subacromial space. A lateral viewing portal is then established, followed by a complete subacromial bursectomy. Given the frequent association between bursal-sided tears and subacromial impingement, a routine arthroscopic acromioplasty is performed using a motorized burr and cutting block technique. An additional anterosuperior portal is created just off the acromial border for suture anchor placement and suture management. After careful debridement of the rotator cuff footprint, a 4.5-mm suture anchor (SwiveLock; Arthrex, Naples, FL) is inserted for tendon fixation (Fig 2A). Then, a No. 1 polydioxanone (PDS) suture (Johnson & Johnson, Edinburgh, Scotland) is introduced percutaneously using an 18-gauge spinal needle, which is passed through the healthy remnant of the supraspinatus tendon into the glenohumeral joint under direct arthroscopic visualization (Fig 2 B and C). The head end of the PDS suture is retrieved through the anterior portal (Fig 2D). Next, a suture hook is introduced through the anterolateral portal into the subacromial space and directed from superior to inferior, penetrating the full thickness of the supraspinatus tendon into the glenohumeral joint (Fig 2E). A second PDS suture is passed in the same manner, and its head end is also retrieved through the anterior portal. One limb of the anchor suture is then tied to the tail end of the first PDS suture. By pulling the head end of this PDS suture, the anchor suture is shuttled through the healthy tendon remnant and retrieved from the anterior portal. Subsequently, the anchor suture is tied to the head end of the second PDS suture. By pulling the tail end of the second PDS suture, the anchor suture is further shuttled through the full thickness of the tendon and again retrieved through the anterior portal (Fig 2F). This process is repeated for the remaining sutures, allowing secure passage of the sutures through both the articular- and bursal-sided tendon fibers while minimizing damage to the healthy tissue (Fig 2G). Pilot holes are then prepared at the lateral edge of the greater tuberosity, and suture strands are secured using PushLock anchors (Arthrex) (Fig 2H). Figure 3 is a schema of the surgical procedure. A follow-up MRI scan at 3 months postoperatively shows satisfactory tendon-to-bone healing. The patient has regained nearly normal shoulder range of motion, resumed active exercises, and reported good functional recovery (Fig 4).

Postoperatively, patients are immobilized in a sling with an abduction pillow for the first 6 weeks. During the initial 6 weeks, only passive range-of-motion exercises are permitted, including Codman's exercises, grip strengthening, and isometric scapular stabilization. Gentle active range-of-motion exercises commence at 6 to 8 weeks, with deltoid and biceps strengthening introduced at 8 to 10 weeks. From weeks 10 to 12, patients aim to regain full, pain-free motion while continuing scapular stabilization and beginning rotator cuff strengthening. Finally, beginning in week 12, strengthening is advanced and sport-specific drills are incorporated, emphasizing flexibility and motion velocity.

## Discussion

Determining the optimal arthroscopic approach to restore the rotator cuff footprint while promoting tendon healing remains an area of active investigation. Current debates focus on whether simple in situ repair is sufficient for bursal-sided partial-thickness tears or whether tear completion is necessary. In situ repair may risk leaving a residual intratendinous defect, potentially leading to persistent pain and delayed rehabilitation.^7^^,^^8^ Additionally, disruption of healthy articular-sided fibers alters native tendon tension and impairs hematoma containment at the tendon-bone interface, potentially compromising healing, as shown by Shin^13^ through MRI evaluations. A systematic review and meta-analysis revealed comparable clinical outcomes between in situ repair and tear completion for Ellman grade 3 bursal-sided tears.^6^ However, experimental studies suggest enhanced healing with complete repair techniques compared with in situ methods.^14^ These findings highlight the need for a technique that combines the benefits of preserving intact fibers with the mechanical advantages of full-thickness repair. Our proposed nondisruptive full-thickness repair technique uses a suture-bridge configuration, preserving the articular-sided fibers while reapproximating the bursal-side layer to the footprint. This strategy maintains native tendon tension, minimizes tissue trauma, and promotes a favorable biological environment for tendon-to-bone healing.

Previous studies have also attempted full-thickness repair techniques for bursal-side PTRCTs. Kim et al.^12^ described suture passage through the articular cuff via Neviaser and superior-medial portals, which is technically demanding and less accessible for inexperienced surgeons. Another technique described by Kim et al.^11^ uses mattress sutures without medial anchors but is best suited for small tears with good bone quality owing to a higher risk of anchor pullout. Although the technique described by Yoo et al.^9^ offers a practical solution for remnant preservation, it causes relatively extensive damage to the intact tendon fibers, potentially altering the physiological tension on the articular-sided portion of the cuff. In contrast, our technique minimizes damage, maintains better tension balance, and is reproducible even for beginners. Nonetheless, if the tear involves nearly the full thickness or the remaining fibers are of poor quality, tear completion and repair may be preferable to preserve function and healing potential.^15^

Our technique has several limitations. First, clinical outcome data are lacking. Future studies are necessary to evaluate postoperative tendon healing, retear rates, and patient-reported outcomes to validate the efficacy of this approach. Second, frequent switching between the subacromial and glenohumeral spaces may increase the surgical time. In addition, the development of advanced suture-passing devices could help streamline the technique. Pearls and pitfalls of every step of this technique are listed in Table 1.

**Table 1:** Advantages and Disadvantages

Advantages
This technique preserves the integrity of the anatomic footprint compared with tear completion.
A greater tendon-bone contact area is achieved with a double-row technique compared with a single-row method.
Maintenance of proprioceptive function may be achieved, as the remnant tissue may contain mechanoreceptors, potentially improving postoperative neuromuscular control.
Disadvantages
Switching between the subacromial and glenohumeral spaces may increase the surgical time.
This technique places higher demands on surgical skill and experience, as precise suture passage through both the remnant and mobilized tendon is required to achieve a durable repair.
An additional anterior working portal is required, which may increase soft-tissue trauma, operative time, and potential risk of neurovascular injury.

Our technique facilitates full-thickness repair of bursal-sided partial rotator cuff tears while preserving intact articular-sided fibers. By minimizing unnecessary tissue disruption and maintaining native tendon biomechanics, this tendon-sparing approach may enhance biological healing and clinical outcomes, warranting further investigation.

## Disclosures

The authors declare the following financial interests/personal relationships which may be considered as potential competing interests: Q.L. reports that article publishing charges were provided by the Natural Science Foundation of China and Natural Science Foundation of Jiangsu Province. All other authors (Y.D., Y.Z., J.Q., Q.J., D.C.) declare that they have no known competing financial interests or personal relationships that could have appeared to influence the work reported in this paper.

## Supplementary Data

Video 1 Main steps of surgical procedure. After administration of a preoperative brachial plexus block and induction of general anesthesia, a 45-year-old male patient with a right-sided bursal-side partial-thickness rotator cuff tear is positioned in the lateral decubitus position. Standard posterior and lateral portals are established, and diagnostic arthroscopy is performed. After confirmation of intact articular fibers, subacromial bursectomy and acromioplasty are performed to improve visualization and reduce impingement. A polydioxanone (PDS) suture is passed percutaneously through the healthy full-thickness tendon using a spinal needle and retrieved through the anterior portal. A suture hook is then used to pass a second PDS suture, and its head end is retrieved through the anterolateral portal. One limb of the anchor suture is shuttled through the tendon remnant by tying it to the PDS suture. The remaining anchor sutures are passed in the same manner. Finally, the sutures are secured with PushLock anchors in a double-row configuration, preserving medial tissue integrity and optimizing healing. The final fixation is inspected arthroscopically to confirm secure repair.
